# Bone marrow stromal cells inhibit caspase-12 expression in rats with spinal cord injury

**DOI:** 10.3892/etm.2013.1201

**Published:** 2013-07-04

**Authors:** WEI LIU, YUEMING DING, XIAOMING ZHANG, LINLIN WANG

**Affiliations:** 1 Department of Prosthodontics, Stomatology Hospital, College of Medical Sciences, Zhejiang University, Hangzhou, Zhejiang 310006, P.R. China; 2 School of Medicine and Life Sciences, Zhejiang University City College, Hangzhou, Zhejiang 310015, P.R. China; 3 Department of Basic Medical Sciences, School of Medicine, Zhejiang University, Hangzhou, Zhejiang 310058, P.R. China

**Keywords:** spinal cord injury, bone marrow stromal cells, caspase-12, endoplasmic reticulum stress

## Abstract

The mechanisms underlying the potentially beneficial effect of bone marrow stem cells (BMSCs) on spinal cord injury (SCI) are unknown. Therefore, the aim of the present study was to explore the protective effect of BMSCs in rats with SCI. A total of 45 adult male Sprague-Dawley rats were randomly divided into three groups; the SCI group (n=15), the BMSC group (n=15) and the sham-operation group (n=15). In the SCI and BMSC treatment groups, a modified Allen’s weight-drop technique was used to induce SCI. The BMSC treatment group received an injection of BMSCs using a microneedle into the epicenter of the spinal cord 24 h after injury. Rats in the sham-operation group were not subjected to SCI; however, the corresponding vertebral laminae were removed. Seven days after transplantation, a rapid recovery was observed in the Basso, Beattie and Bresnahan (BBB) scores of the BMSC treatment group, whereas the BBB scores in the SCI group remained low (P<0.05). Caspase-12 expression in the SCI group was increased compared with that in the sham-operation group, whereas caspase-12 expression was attenuated 24 h after transplantation in the BMSC treatment group (P<0.05). In conclusion, the transplantation of BMSCs may improve locomotor function and attenuate caspase-12 expression following SCI. Therefore, it is likely to be an effective strategy for preventing severe injury of the spinal cord.

## Introduction

Spinal cord injury (SCI) often causes life-long disability. Therefore, it is important to gain an understanding of the pathophysiology underlying SCI in order to develop clinical treatment strategies (1,2). Typically, there are two phases of SCI; the primary injury, in which mechanical impact is afflicted directly on the spine, and the secondary injury, which involves a complex cascade of molecular events, including disturbances in ionic homeostasis (3), local edema, vascular abnormalities, ischemia-reperfusion, glutamate excitotoxicity and inflammatory responses (4–6). Previous studies have indicated that, following SCI, stress responses of the endoplasmic reticulum (ER) drive neurons and oligodendrocytes towards apoptosis (7). An ER-resident caspase, caspase-12, has been shown to mediate apoptosis signaling induced by ER stress (8). Furthermore, activation of the unfolded protein response (UPR) upregulates the expression and activity of caspase-12 and may be important in neuronal cell death in ischemic brain injury (9). It has also been observed that the activation of caspase-12 is involved in the apoptosis of spinal cord neurons and oligodendrocytes, which is induced by oxygen-glucose-serum deprivation/restoration (10,11). Therefore, the inhibition of caspase-12 expression following SCI may potentially be used as a therapeutic target for neuron protection.

Bone marrow stromal cells (BMSCs) have been identified as potential candidates for the treatment of SCI (12). BMSCs are a population of heterogeneous mesenchymal cells in the bone marrow consisting of pluripotent mesenchymal stem cells and murine BMSCs. Under suitable conditions, they may be propagated *in vitro* for up to 50 passages with no signs of malignant transformation (13). The transplantation of BMSCs into SCI rats to promote axonal regeneration, reduce lesion size and improve functional outcome has been reported (14,15). At present, the beneficial effects of BMSCs in several models of CNS injury are considered to be due to the release of trophic factors and the activation of endogenous survival signaling pathways via secreted soluble factors in neurons and oligodendrocytes, as opposed to a result of neuronal or glial differentiation (16). However, the exact mechanisms underlying the protective effects of BMSCs in SCI remain unknown.

In the present study, BMSCs were injected into the spinal cords of modified Allen’s weight-drop SCI model rats in order to investigate their effect on apoptosis and the expression of caspase-12. The aim of this study was to investigate the protective role of BMSCs in the rats with SCI and to attempt to develop an effective clinical method of treatment for SCI.

## Materials and methods

### BMSC culture and induction of SCI in the rat model

Primary rat BMSCs were isolated and characterized using methods described in our previous work (17). In brief, the tibias and femurs were dissected from Sprague-Dawley adult male rats under sodium pentobarbital (40 mg/kg, i.p.) anesthesia. Rats were purchased from the Experiment Animal Center of Zhejiang University. After removing the end of each bone, 5 ml BMSC culture medium, consisting of α-MEM (Gibco-BRL, Carlsbad, CA, USA) supplemented with 20% fetal bovine serum and antibiotics, was injected into the central canal of the bone in order to extrude the marrow. Whole marrow cells were extracted and cultured at a density of 5–10×10^5^ cells/cm^2^ in BMSC culture medium. The nonadherent cells were removed after 24 h by changing the medium and the medium was changed every other day until the cells became confluent. Subsequently, when the cells were nearly confluent, they were detached by trypsin and serially subcultured in the ratio 1:3, passaged three times and washed twice with PBS to a concentration of 10^6^ cells/100 μl for transplantation.

A total of 45 Sprague-Dawley rats were randomly divided into three groups (the sham-operation, SCI and BMSC treatment groups; n=15 in each group). Under sodium pentobarbital (40 mg/kg, i.p.) anesthesia, the vertebral columns of the rats were exposed and a laminectomy at level T10 was carried out. Subsequently, a contusion injury was produced using a weight-drop device in the SCI and BMSC treatment groups. A weight of 10 g was dropped from a height of 50 mm onto the exposed spinal cord and the impounder was left for 20 sec before withdrawal in order to produce a moderate contusion. Rats in the sham-operation group underwent the same surgical procedure but without SCI. Twenty-four hours after the operation, 5 μl labeled BMSCs were injected using an electrode microneedle into the epicenter of the injured spinal cords of the BMSC treatment group rats, while the same quantity of PBS was injected into rats of the SCI and sham-operation groups. Hind limb locomotor function was assessed using the Basso, Beattie and Bresnahan (BBB) locomotor rating scale at 24 h and 7 days after transplantation (18). The rats were sacrificed for subsequent pathological, immunohistochemical and quantitative (q)PCR analyses. All animal experiments were conducted with the approval of the Animal Care Committee of the School of Medicine, Zhejiang University (Hangzhou, China).

### Immunohistochemical assay

Five rats per group were re-anesthetized with sodium pentobarbital (60 mg/kg, i.p.) and perfused with 4% paraformaldehyde in phosphate-buffered saline 24 h after transplantation. The lesion epicenter (4 mm) of the spinal cord was removed, post-fixed in the same fixative for 24 h and prepared for cryostat sectioning.

DAB staining was performed on the cryostat sections (thickness, 8 μm). Briefly, cryostat sections were first washed in PBS and incubated with 1% hydrogen peroxidase (H_2_O_2_) to block the non-specific reactivity of endogenous peroxidase. After washing in phosphate buffered saline (PBS), the sections were treated for antigen retrieval with 10.2 mmol/l sodium citrate buffer (pH 6.1) for 20 min at 95°C, followed by incubation in medium of PBS with an anti-caspase-12 primary antibody (1:150; Santa Cruz Biotechnology, Inc., Santa Cruz, CA, USA) plus 1% BSA at 25°C for 2 h. Control sections were incubated in PBS plus 1% BSA. After washing, the sections were incubated for 1 h at 25°C in a biotinylated goat-anti-mouse secondary antibody (diluted in 1:200 in PBS, Boster Biological Technology, Ltd., Wuhan, China), and subsequently the sections were incubated with a horseradish peroxidase (HRP)-conjugated avidin-biotin complex (ABC) for 20 min, and the staining was visualized with 0.05% DAB plus 0.3% H_2_O_2_ in PBS. The sections were also stained with hematoxylin for 1 min, and the stained sections were dehydrated with a graded series of ethanol and xylene before using cover slips. Images of the ventral area of the spinal cord were captured (magnification, ×40) using a digital camera attached to a microscope (Nikon E600, Nikon Company, Tokyo, Japan). Total numbers of caspase-12-positive cells were manually counted in 10 fields in a section around the injured area. The results were expressed as the average number of caspase-12-positive cells per field in each group.

### qPCR assay

The remaining 5 rats per group were re-anesthetized and decapitated for RNA sample preparation. The lesion epicenter corresponding to one intervertebral segment at the T10 cord level was removed (~4 mm) and total RNA was obtained using an RNA extraction kit (Qiagen, Hilden, Germany) according to the manufacturer’s instructions. The reaction mixture used for qPCR (40 μl) contained 4 μl cDNA, 35.2 ml SYBR-Green PCR mix, 5 units (0.5 μl) Taq DNA polymerase and 0.3 μl 20 pmol/ml caspase-12 primer. The cDNA was denatured at 94°C for 3 min. The template was amplified for 40 cycles (denaturation at 94°C for 10 sec, annealing at 57°C for 30 sec and extension at 72°C for 30 sec), prior to detecting fluorescence at 72°C. Meanwhile, primers for housekeeping gene glyceraldehyde-3-phosphate dehydrogenase (GAPDH) were used in PCR to amplify GAPDH (forward: 5′-AGTTCAACGGCACAGTCAAG-3′ and reverse: 5′-TACTCAGCACCAGCATCACC-3′) as an internal control for caspase-12 (forward: 5′-CACTGCTGATACAGATGAGG-3′ and reverse: 5′-CCACTCTTGCCTACCTTCC-3′). Fold-change in gene expression was estimated using the comparative C(T) method (19).

### Statistical analysis

All values are presented as the mean ± SD. Statistical comparisons in all groups were made using one-way analysis of variance (ANOVA). A probability of 95% (P<0.05) was considered to indicate a statistically significant difference. Fold-changes in gene expression were estimated using the CT comparative method normalizing to GAPDH CT values and relative to control samples as follows: ΔC_T_ = C_T_ caspase-12 - C_T_ GAPDH; ΔΔC_T_ = ΔC_T_ - ΔC_T_ control; fold difference = 2^−(ΔΔCT)^.

## Results

### BMSC transplantation improves limb locomotion

**Figure 1:**
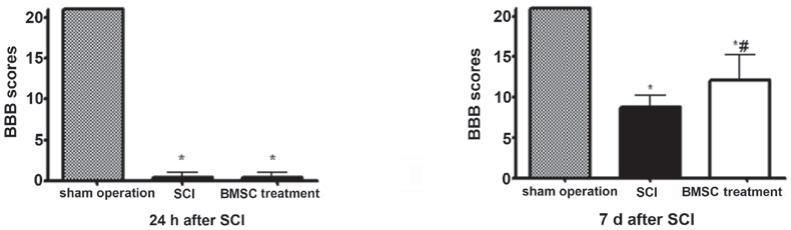
BBB scores in the three groups at 2 h and 7 days after transplantation. Scores were significantly reduced in the SCI and BMSC treatment groups compared with those in the sham-operation group 24 h and 7 days after transplantation (^*^P<0.05). Scores were significantly increased in the BMSC treatment group compared with the SCI group 7 days after transplantation (^#^P<0.05). BBB, Basso Beattie and Bresnahan; BMSC, bone marrow stem cell; SCI, spinal cord injury.

Rats in the SCI and BMSC treatment groups exhibited marked bilateral hind limb paralysis, with no movement or only slight movement of the joints, when observed during open-field walking. By contrast, all rats in the sham-operation group were able to walk normally 24 h after injury. The animals were observed for 7 days post-transplantation in order to measure their locomotor activity according to the BBB scale. During the 7 days, the degree of recovery of locomotor function, to a plateau below the range of 10 points, was observed in the SCI group compared with rats in the BMSC treatment group, which exhibited greater recovery (BBB scores >10, P<0.05; Fig. 1).

### Immunohistochemistry and qPCR assay

**Figure 2:**
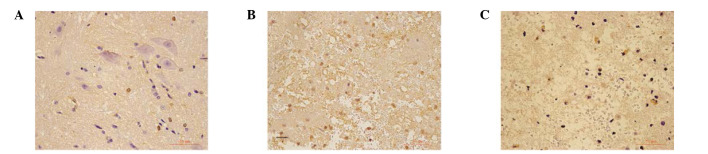
Caspase-12 immunohistochemical DAB staining of spinal cord sections 24 h following surgery in the (A) sham-operation, (B) spinal cord injury (SCI) and (C) BMSC-treated rats (magnification, ×400). BMSC, bone marrow stem cell; DAB, diaminobenzidine.

Cells that stained positive for caspase-12 exhibited buff-colored granules when stained with diaminobenzidine (DAB; Fig. 2). Caspase-12 was located at the neurons and oligodendrocytes. There were few caspase-12 positive cells in the sham-operation group. However, 24 h after transplantation, the neurons and oligodendrocytes showed an increased caspase-12-positive cell number (16.8±3.5/field) when compared with the sham operation rats (5.3±0.7/field), whereas the number of caspase-12-positive cells was decreased in the BMSC treatment rats (11.2±2.6/field, P<0.05) 24 h after transplantation.

**Figure 3:**
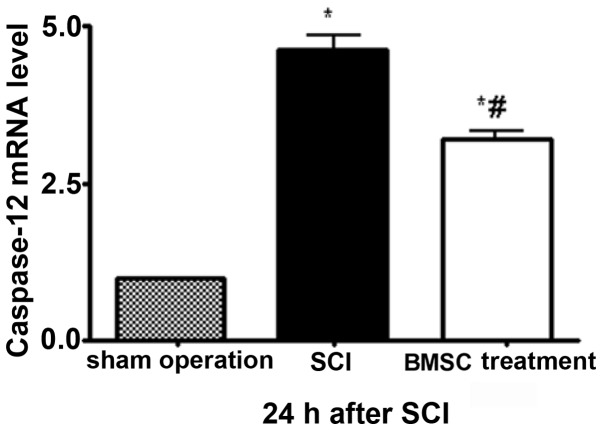
Caspase-12 mRNA expression levels in the three groups 24 h after transplantation. Levels of caspase-12 expression were significantly increased in the SCI and BMSC treatment groups compared with those in the sham-operation group (^*^P<0.05). Expression levels were significantly reduced in the BMSC treatment group compared with the SCI group (^#^P<0.05). BMSC, bone marrow stem cell; SCI, spinal cord injury.

Moreover, compared with the sham-operation rats, the mRNA expression levels of caspase-12 increased in the SCI group 24 h after SCI, whereas BMSC treatment reduced the caspase-12 mRNA expression at the same time-point after transplantation (P<0.05; Fig. 3).

## Discussion

An understanding of the pathophysiology underlying SCI is important for guiding research efforts and developing clinical treatment strategies (20,21). Upregulation of caspase-12 is important in ER stress-induced cell death in brain ischemia (22), Parkinson’s disease (23), Alzheimer’s disease (24) and SCI (11). Previous studies have revealed that SCI triggers ER stress responses and that therapeutically reducing ER stress-induced SCI may be an efficient method of treatment for ischemic SCI (25,26). Consistent with previous investigations (11,27), the present study also demonstrated an increased production of the pro-apoptotic factor caspase-12.

BMSCs, also known as mesenchymal stem cells, are a mixed cell population that includes stem and progenitor cells. These cells are easily obtained, available for autologous transplantation, immune-privileged to allogeneic cells and able to migrate to areas of inflammation (28,29). There is increasing evidence to suggest that transplanted stem cells operate as ‘small molecular factories’; following brain attack, they secrete neurotrophins, growth factors and other supportive substances that may have continual therapeutic benefits in brain ischemia (30). Since treating SCI involves repairing the initial injury of severed fiber tracts, as well as fighting widespread secondary damage, stem cell therapy may aid the regeneration of damaged tissue in the injured area via provision of new cells. Furthermore, stem cells may counteract factors in the lesion environment that inhibit axonal regeneration (31). The results of the present study demonstrated that BBB scores recovered rapidly following BMSC transplantation and the highest density of BMSCs was observed surrounding the SCI. Pathological examination also revealed that lesioned tissues contained numerous cells, milder scar formation and reduced cavities. Caspase-12 is known to have a negative impact on SCI (11,27). Although the caspase-12 enzyme was not analyzed via western blotting, this study provided direct evidence that stem cells suppress caspase-12 expression. These results suggested that BMSCs may facilitate the recovery of neurons and oligodendrocytes from SCI by attenuating caspase-12 expression, thus aiding remyelination and functional recovery processes (32,33). Further investigation is required to elucidate the direct mechanism underlying the anti-apoptotic effect of BMSCs on neurons and oligodendrocytes.

In conclusion, the present study provided further insight into treatment methods for SCI by demonstrating that BMSC transplantation attenuates the expression of caspase-12. However, successful development of a BMSC therapy for SCI requires an improved understanding and further biochemical assessments of the long-term positive effects of BMSCs.
